# Magnetoelastic Ribbons as Vibration Sensors for Real-Time Health Monitoring of Rotating Metal Beams

**DOI:** 10.3390/s21238122

**Published:** 2021-12-04

**Authors:** Georgios Samourgkanidis, Dimitris Kouzoudis

**Affiliations:** Department of Chemical Engineering, University of Patras, 26504 Patras, Greece; G.Samourgkanidis@gmail.com

**Keywords:** Metglas ribbons, magnetoelastic materials, vibration sensors, rotating cantilever beams, bending modes

## Abstract

In the current work, magnetoelastic material ribbons are used as vibration sensors to monitor, in real time and non-destructively, the mechanical health state of rotating beam blades. The magnetoelastic material has the form of a thin ribbon and is composed of Metglas alloy 2826 MB. The study was conducted in two stages. In the first stage, an experiment was performed to test the ability of the ribbon to detect and transmit the vibration behavior of four rotating blades, while the second stage was the same as the first but with minor damages introduced to the blades. As far as the first stage is concerned, the results show that the sensor can detect and transmit with great accuracy the vibratory behavior of the rotating blades, through which important information about the mechanical health state of the blade can be extracted. Specifically, the fast Fourier transform (FFT) spectrum of the recorded signal revealed five dominant peaks in the frequency range 0–3 kHz, corresponding to the first five bending modes of the blades. The identification process was accomplished using ANSYS modal analysis, and the comparison results showed deviation values of less than 1% between ANSYS and the experimental values. In the second stage, two types of damages were introduced to the rotating blades, an edge cut and a hole. The damages were scaled in number from one blade to another, with the first blade having only one side cut while the last blade had two side cuts and two holes. The results, as was expected, show a measurable shifting on the frequency values of the bending modes, thus proving the ability of the proposed magnetoelastic sensors to detect and transmit changes of the mechanical state of rotating blades in real time.

## 1. Introduction

The accurate assessment of the mechanical health of rotating machinery used in various aspects of industry (factories, power plants, airplanes, ships, automobiles, etc.) is one of the most difficult challenges in modern industrial technology due to the significant financial losses and security risks. As sensor and automation technologies progress, more and advanced sensor devices are used to monitor the mechanical health state of the machinery and determine the root cause and the severity of the faults. In general, a mechanical structure can be maintained in three different ways: after failure, preventive failure, and predicted maintenance [1]. In the first case, the equipment is maintained after a major failure so it is unable to continue its operation. This method generally necessitates high maintenance costs and poses a risk to personal safety. The second case (preventive failure) is based on the wear law of equipment parts and treats the part failure as a function of time. While this method is planned and preventative in certain aspects, it can be fairly costly when the parts are free of faults. Finally, the predicted maintenance method is based on the equipment’s current condition of operation, and the approach employs various types of sensors to determine the machinery’s health status in real time. Health monitoring with advanced measurement techniques is typical of this method, and the advantages are numerous (improved machine efficiency, dependability, availability, durability, reduced maintenance costs, programs to improve safety and the environment, etc.).

The measurement of fault signals is a crucial foundation for rotating machinery health monitoring and there is a range of signals (light, sound, temperature, etc.) that can be used for this purpose. One of these signals is vibration, and is the most extensively utilized way of monitoring [2,3,4,5,6,7,8,9,10,11,12,13]. In general, a vibration signal contains a large amount of data related to the material and structure of the machinery, and with a comprehensive analysis it can reveal the degree and magnitude of the fault. Another significant benefit of vibrating signals is that they may be used easily and reliably in real-time mechanical health evaluation procedures of a structure, as long as they are combined with the suitable damage detection methodology [14,15,16,17,18]. Vibration sensors are used to detect vibration signals and are classified as either contact or noncontact. The most common contact vibration sensors are the piezoelectric, fiber Bragg frating (FBG), and interferometric optical fiber vibration sensors, while the most common noncontact vibration sensors are the eddy current sensor, reflective optical fiber sensor, fiber tip-timing sensor, noncontact FBG vibration sensor, and laser Doppler vibrometer.

Sunar and Al-Bedoor [19] studied, experimentally and numerically (by finite element method), the suitability of a piezoelectric (PZT) sensor for monitoring rotational blade vibrations. The piezoelectric material was inserted directly between the rotor and the blade root, and the output signal was delivered wirelessly using a Binsfeld transmitter that sent the signal to a stationary receiver continuously. Based on both FEM and experimental results, they concluded that the root-embedded PZT sensor may be efficiently used for blade vibration measurements in a wide range of applications. Vilchis-Rodriguez et al. [20] demonstrated the use of FBG accelerometers to monitor, in a wide band (<1 kHz), the vibration of a wound rotor induction generator in a wind turbine. They compared the FBG accelerometer to a commercial piezoelectric vibration sensor, and the experimental results showed that the FBG sensor produced a greater output signal in the frequency range of 600–1000 Hz.

He et al. [21] proposed a vibration measurement technique which utilized an optical fiber distributed sensing system. On both ends of the sensing fiber with a temporal difference, two acoustic optical modulators were used to generate narrow and wide pulses, respectively. Wide pulses interfere with reference light, and narrow pulses generate Rayleigh back-scattering light to locate the vibration point. The rapid break of pencils near the fiber loop was measured to model the high-frequency responses of cracks in civil constructions. The experimental results reveal that at 1150 m sensing distance, 5 m spatial resolution and up to 6.3 MHz frequency response with 50 ns pulse width may be accomplished. Devillez and Dudzinski [22] presented a method to measure the vibration of a lathe machine using eddy current sensors. Three specific signals were investigated, which were obtained during the stable, quasi-unstable, and unstable tests. The results suggested that the eddy current sensor may be used to detect tool micromovement during the machining process. Zhang et al. [23] measured the lubricant film thickness of sliding bearings using reflective optical fiber sensors. During slow rotations, the experimental results revealed constant lubricating layer thicknesses, thus showing a linear relationship between sensor output and film thickness.

Chen et al. [24] investigated the concept of measuring the vibration of rotating blades using four tip-timing fiber sensors. The frequency, amplitude, and other related parameters of the recorded vibration signal were extracted using principal component analysis (PCA). The collected data were evaluated in order to validate the proposed method, and the experimental findings show that it works. Li et al. [25] suggested a noncontact vibration sensor, utilizing a hybrid FBG sensor to measure the vibrations of a rotating shaft. The key components of the suggested sensor were a neodymium iron boron permanent magnet, a movable head, a circular diaphragm, a fixed screw, and an FBG sensor. Experimental results show that the resonant frequency of the sensor was about 1500 Hz and the working band ranges within 0–1300 Hz, which was consistent with the simulation analysis result. Neumann et al. [26] presented vibration measurements on a turbine compressor using a laser Doppler distance sensor (LDDS). They measured simultaneously the radial blade expansions and the circumferential blade deflections, as well as the circumferential velocities of the rotor blade tips, and the results show an agreement of the recorded vibration frequencies and the vibration increase of the blades, before stall, with the measurement results of a commercial capacitive blade tip timing system.

This work is a continuation of our prior research [27,28,29,30] and takes us a step closer towards understanding and using magnetoelastic ribbon materials as vibration sensors for health monitoring applications. The novelty in this study is that the proposed magnetoelastic vibration sensor is used for noncontactless and nondestructive health monitoring of a rotating mechanical structure, rather than a stationary one, and is the proof of concept in this direction. Additionally, the experiment’s setup and execution have been designed in such a way that the health of the rotating structure can be monitored in real time. In general, the magnetoelastic effect (or Villari effect) occurs when mechanical stress is applied to certain ferromagnetic materials, causing their magnetic susceptibility to vary, while magnetostriction is the inverse effect, and is the continuous deformation of the ferromagnetic material under the application of a magnetic field. A property that determines whether or not a ferromagnetic material is magnetoelastic is the magnetoelastic coupling coefficient k [31,32], which is equal to the ratio of conversion of the magnetic to the elastic energy (0 < k < 1). The Metglas alloy 2826 MB is one of the most well-known metallic glasses, and it was used in this investigation. It is an iron–nickel alloy with high corrosion resistance that is typically formed into thin foils or ribbons. It is extremely flexible and strong due to its geometry and elasticity, and it has many applications in sensing research [33,34,35,36,37].

## 2. Materials and Methods

**Figure 1:**
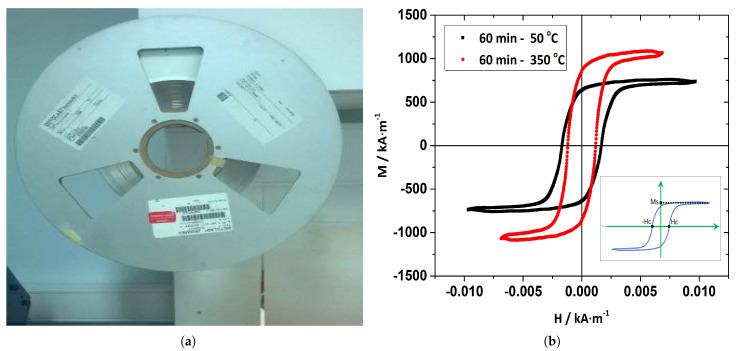
(**a**) Metglas 2826 MB ribbon spool. During the material production process, the spool length usually reaches hundreds of meters. (**b**) Hysteresis loop of the Metglas 2826 MB in two different temperatures.

Ribbons of magnetoelastic material known as Metglas 2826 MB are used in this work as vibration sensors in order to detect and transmit the vibration state of the rotating beams. This is a nickel–iron-based, soft, ferromagnetic alloy material that is usually produced in the form of a long thin ribbon (Figure 1a), by quenching methods, and is widely used in transformer applications due to the high permeability, linearity, and low eddy currents. Table 1 summarizes the physical and geometric properties of the ribbon as given by the manufacturer. According to this table, the properties of interest, in order to use these materials in sensing applications, are numbers 7, 8, and 9. The property number 7, which is the annealed saturation induction, is directly connected to the strength of the output signal of the sensor and depends, to a large extent, on the temperature at which the material is annealed. The Figure 1b graph presents the hysteresis loops (material magnetization M versus the applied magnetic field H) of the Metglas 2826 MB for two different annealing temperatures, within the time range of 60 min. It is clear that the saturation magnetization M_*s*_ (the maximum value of the magnetization) increases at higher temperatures and that the coercivity field H_*c*_ (the value where the loop crosses the horizontal axis) decreases. Thus, the material becomes a “softer ferromagnet”, improving sensing capacity.

Properties number 8 and 9, which are the Curie and crystallization temperatures, are directly related to the operating temperature range of the sensor. In the case of the Curie temperature, the sensor material moves from the ferromagnetic to the paramagnetic state with increasing temperature, thus altering the output signal without permanent effects, while in the case of the crystallization temperature, the output signal is altered due to the change in the crystalline structure of the material with permanent results. Annealing above the crystallization temperature significantly affects the performance of the material as a sensor, as after a certain period of time, the signal strength of the sensor is almost eliminated.

**Figure 2:**
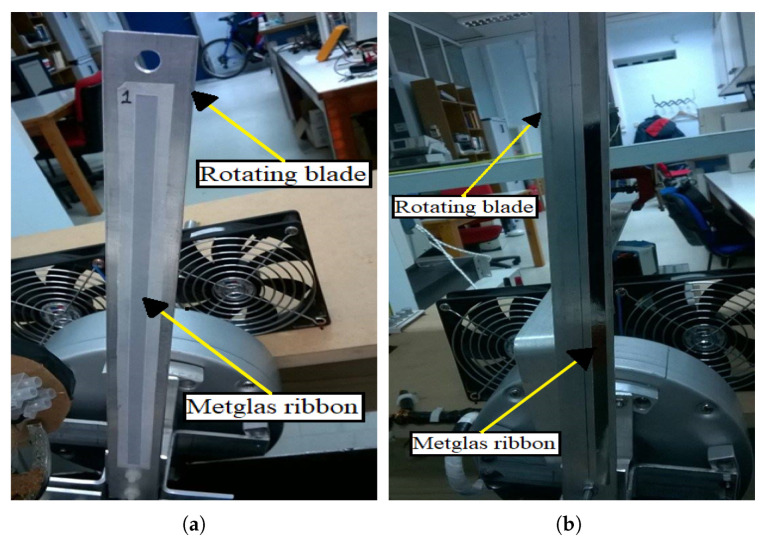
(**a**) A piece of a Metglas 2826MB ribbon attached to a rotating blade with double-sided tape. (**b**) The same rotating blade with the excess tape was stripped thoroughly using a strong plastic blade to prevent the beam from scratching.

Figure 2 shows a single piece of a annealed Metglas ribbon attached to a rectangular aluminum beam blade. The length of the ribbon is 25 cm and the length of the blade is 30 cm. In general, the proper ribbon size is determined by two factors: the size of the spinning mechanical part, and the type of detection coil used in the signal detection method, which also includes various signal amplification methods. However, the majority of the time, the decision is made through trial and error. In our case, a 25 cm ribbon and a 625-thread coil were sufficient to record the vibration signal without any amplification. The aluminum blade is mounted on a single-phase electric motor with a fixed rotor and a rotating stator. Later, in the experimental section (Section 3), the motor setting and the rotating blades are described in more detail. The prepared ribbon is attached on one side of the blade using a 3M^TM^VHB^TM^ 4930 double-sided tape, of which physical properties are presented in Table 2, as given by the manufacturer. It should be noted at this point that, while it is difficult for the ribbon to detach due to its low mass and, thus, inertia, detachment can occur in practice. As a result, it is critical to select adhesives that are compatible with the surfaces on which they are used. In this case, the adhesive used interfaces well with metals where high dynamic stresses are involved, and it perfectly fits the experimental process of the work. Finally, the rotating blades used are made of 6063 aluminum alloy, and their dimensions were set using a CNC machinery in a workshop. Table 3 presents the physical properties and dimensions of the rotating blades.

## 3. Experimental Setup and Procedure

**Figure 3:**
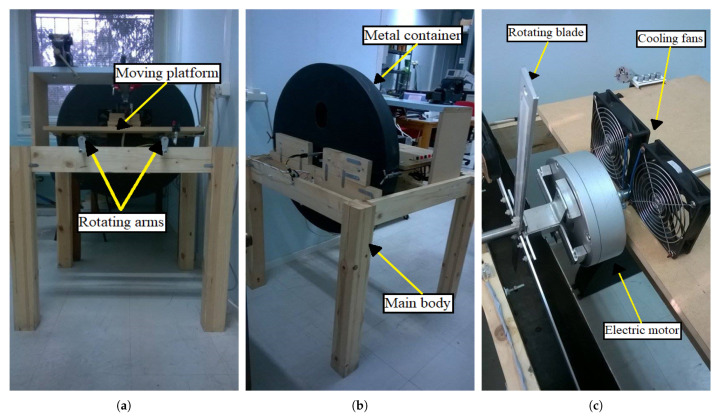
(**a**) Front view of the setup. The moving platform is shifted back and forth on metal rails using two rotating arms. (**b**) Rear view of the setup and the metal container inside of which the mounted, on the motor, blades are rotating, (**c**) View of the electric motor as it is fixed on the moving platform. The blades, mounted on the motor, are also visible inside the half-open metal container.

The experimental setup shown in Figure 3a,b was designed and built to test the hypothesis that magnetoelastic sensors can detect, in situ, the vibrations of a rotating blade, and transmit in a contactless fashion the vibration behavior of the blade. The setup is divided into three main parts, which are the main body, the moving platform, and the metal container. The main body is made of wood and is intended to hold the other two parts firmly during the experiment. The moving platform is also made of wood and is mounted on two metal rails which are attached to the upper side of the main body. The role of the platform is to move back and forth, using two rotating arms, in order to control the distance between the rotating blades and the detection coil. Later, in this section, it is explained how the vibrational signal is detected from the rotating blades. In addition, the moving platform has all the electronic devices integrated on it in order to automate the experimental process. The last part is the metal container, located at the back of the experimental setup. The role of the metal container is twofold: firstly, to keep the experimenter safe from the rotating blades, and secondly, to act as a Faraday cage in order to reduce the electromagnetic noise inside it, as the detection method was performed using coils which are sensitive to electromagnetic noise.

**Figure 4:**
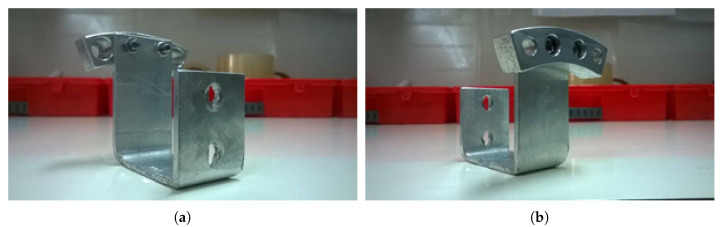
(**a**) Front and (**b**) rear view of the base used to mount the blades on the motor. The Π shape of the base made it easy to close the metal container without the blades resting inside it as they rotated.

Figure 3c shows the electric motor used, along with the mounted blades, on the moving platform. As was mentioned above, the electric motor is a single-phase electric motor with a fixed rotor and a rotating stator. Two stainless-steel tubes are attached at each side of the rotor to fix the motor on the moving platform, and they were also used to pass the motor cables through them to protect them from the rotary motion of the blades. The motor can be set to rotate at three different speeds of 60 rpm, 120 rpm, and 180 rpm. For the purpose of this work, only the highest speed of 180 rpm was used. The two cooling fans were placed in order to dissipate heat away from the motor, as an increase in temperature of up to 60 °C was observed during the experiments. A set of four blades was mounted on the electric motor using a specially shaped base (Figure 4), so that they can be easily inserted into the metal container.

**Figure 5:**
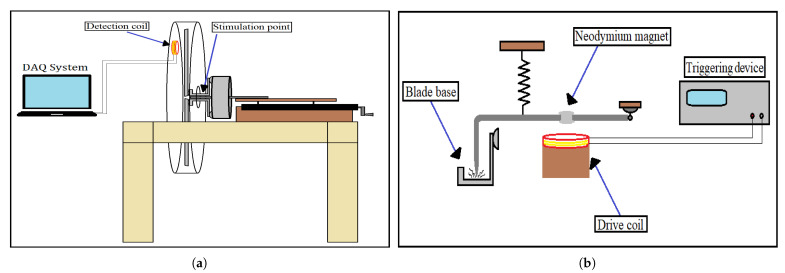
(**a**) Schematic of the experimental setup. A detection coil connected to a laptop was used to detect the vibrations of the rotating blades. (**b**) Schematic of the mechanical stimulation system. The edge point of the Γ-shaped rod excites the stimulation point shown in Figure 5a.

The experimental procedure was performed as follows: A detection coil (with physical and geometrical properties shown in Table 4) was mounted on the back and top of the metal container via a hole drilled for this purpose (Figure 5a). The coil terminals were connected with a special plug to the microphone socket of a laptop, and the measurements were carried out using Matlab software, where an algorithm was created to record and calculate the fast Fourier transform (FFT) spectrum of the signal of each blade separately. The measurements were recorded in situ, with the blades rotating, and the results were extracted within a few seconds. The basic principle of the detection method is the Faraday’s law of induction, and it works as follows: The attached Metglas ribbons on the rotating blades are forced to follow the mechanical oscillations of the blades, and, due to the magnetoelastic character, they change their magnetic state (magnetization) continuously. The change in the magnetic state of the ribbons produces an AC magnetic field which, in turn, induces an EMF voltage to a nearby coil through Faraday’s law of induction.

**Figure 6:**
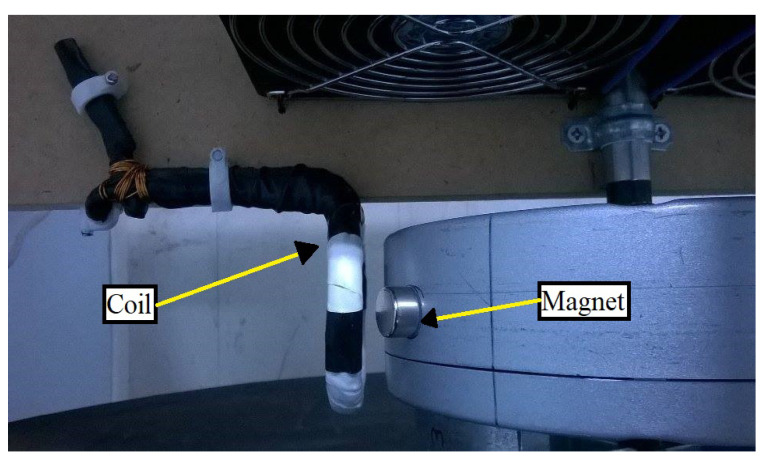
The second set of neodymium magnet and coil. The magnet is fixed to the motor and rotates along with it, passing next to the coil and inducing a voltage on it.

During the first tests, an attempt was made to record the signal solely by the vibrations the blades are forced to make due to their rotation. However, it turned out that the recorded signal was very weak and comparable to the noise level, and any attempt to amplify it and record it failed due to the noise level. To overcome this situation, an automatic mechanical stimulation was designed, as shown in Figure 5b. A Γ-shaped aluminum rod is fixed on one end while the other end, which has the form of a sharp tip, is hanging with the use of a spring over the flat side of the base of the blades. A neodymium magnet fixed on the rod is attracted by a drive coil each time the coil is triggered and forces the tip of the rod to mechanically stimulate the base. This stimulation sets the mounted blade into free vibrations and, as discussed later, it mostly excites the bending, to the easy axis, oscillations of the blade. Finally, as Figure 6 shows, a second set of a neodymium magnet and a coil was used to synchronize the excitation process with the rotation of the blades. As the neodymium magnet is attached to the motor, it rotates with it, and each time it passes near the coil, it triggers an arbitrary waveform generator (TTi—TGA1241). The generator then sends a short pulse to the driving coil (Figure 5b), which attracts the aluminum rod and forces the tip to impact on the base, mechanically exciting both the base and the blades attached to it. The position of the magnet on the motor was set so that the blades are mechanically excited each time they pass directly opposite to the detection coil.

## 4. Results and Discussion

The experimental measurements were performed in two stages. In the first stage, the measurements were performed on rotating blades without damages, and the results were verified using the ANSYS software program, while in the second stage, the measurements were performed by inserting damages to the rotating blades. Regarding the first stage, the experimental procedure is the one described in Section 3.

**Figure 7:**
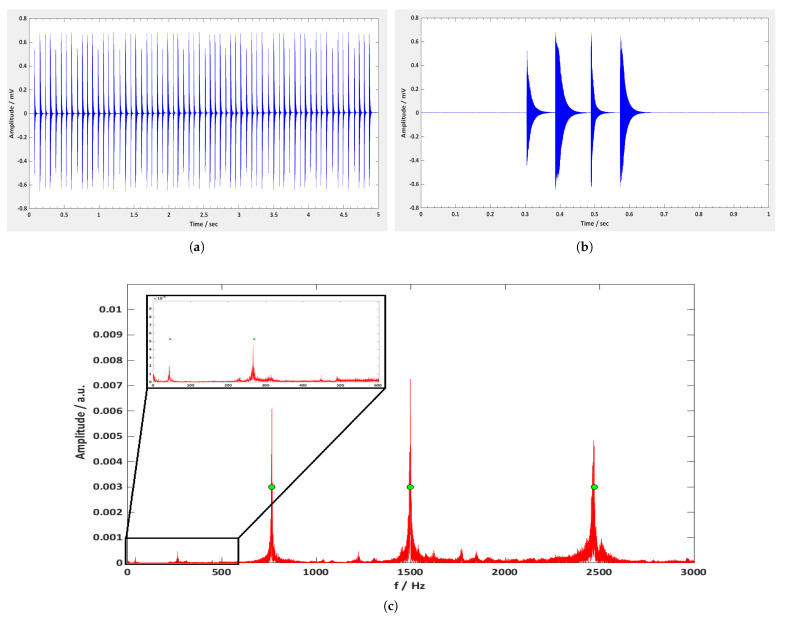
(**a**) Excitation signal of the undamaged rotating blades versus time. (**b**) Excitation signal for one rotation only. (**c**) FFT spectrum of the 1st undamaged rotating blade. The green circles indicate the frequency values of the first 5 bending modes of the undamaged blade as they were calculated by ANSYS modal analysis.

Figure 7a presents the measured data within the time range of 5 s, as they were recorded using the Matlab software program. It can be seen that during the first second there is a total number of 12 peaks in the graph, corresponding to three complete revolutions of the motor, as there are four blades mounted on the motor. This confirms the 180 rpm rotation speed of the motor. Figure 7b shows the same results but for one rotation only. Each peak corresponds to the oscillation signal of each blade, with the first peak being the signal of the blade number 1, the second peak being the signal of the blade number 2, etc. It can be seen that the first peak has the smallest amplitude, something that can also be confirmed in Figure 7a. This is due to the smaller length of the Metglas ribbon attached to blade 1, from which the detection signal is weaker, and this was done to make clear which peak corresponds to which blade, to make it easy to distribute the data per blade using Matlab scripts. Figure 7c presents the FFT spectrum of the recorded signal from the first rotating blade (the blade with the smaller Metglas ribbon). It is clear that within the 3 kHz range of the graphs, there are a number of peaks with different amplitude and width. Below 500 Hz, there are two weak peaks with frequency values of 43.3 Hz and 267.3 Hz, respectively, while above 500 Hz, there are three very high peaks at frequencies of 765.4 Hz, 1498.1 Hz, and 2468.7 Hz, respectively. Since these data were collected from the mechanical oscillations of the first rotating blade, the corresponding FFT peaks should be associated with its natural vibration. To determine these modes, simulations were performed using ANSYS modal analysis.

**Figure 8:**
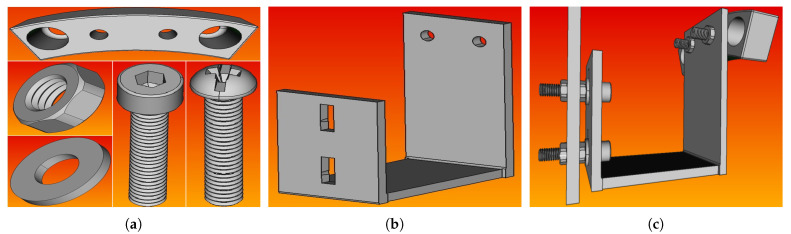
Designed parts of the rotating blade using FreeCAD software program: (**a**) bolts and (**b**) base, (**c**) assembled structure.

**Figure 9:**
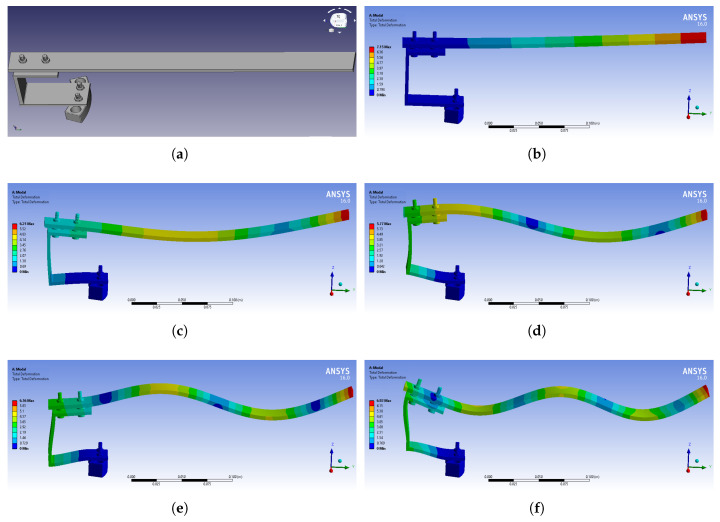
(**a**) View of the assembled rotating blade along with the first 5 bending modes of it on the easy axis, as they were calculated using ANSYS simulations: (**b**) 1st, (**c**) 2nd, (**d**) 3rd, (**e**) 4th, (**f**) 5th.

Figure 8 shows the parts and the assembled structure of the rotating blade, as designed in the FreeCAD software program. The designed model was then introduced into ANSYS modal analysis in order to calculate its free vibration modes. The material properties of each part were introduced using the Engineering Data section of ANSYS modal analysis, and the meshing properties on each part were chosen depending on the structure’s complexity. The bolts were meshed with tetrahedral elements, while the rest of the parts were meshed with hexahedral elements. To combine the parts in one piece, bonded type connections were used. Figure 9b–f present the first five bending modes of the structure to the easy axis, and the frequency values of them are displayed in green circles in Figure 7c. The percentage difference between the ANSYS and the experimental frequency values are shown in Table 5. According to these results, the dominant peaks in the FFT spectrum of Figure 7c correspond to the first five bending modes of the first rotating blade, which does not differ from the other blades, as they are all identical and without damages. In general, the frequency value of each peak is the vibration identity of the rotating blade and it is directly related to the physical and geometrical characteristics of the blade, such as the dimensions of the blade, the modulus of elasticity, the moment of inertia, etc. On the other hand, the amplitude of each peak shows the magnitude of the vibrations, and it is directly related to the energy of the mechanical excitation as well as the size of the sensor.

**Figure 10:**
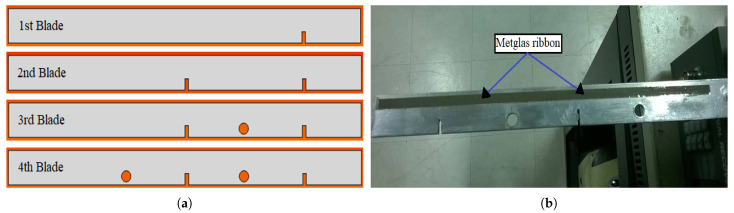
(**a**) Schematic illustration of the type of the damage introduced on each rotating blade. (**b**) The damaged 4th rotating blade along with the attached Metglas ribbon.

The second stage of measurements was performed by introducing various types of damages to the rotating blades. Figure 10a shows, schematically, the type of damages introduced on each blade. The first blade was damaged with a side cut of 1/3 the width of the blade and 2 mm wide, close to the free end of the blade. The second blade was damaged with the same type of cut but at two different locations, one at the middle and one at the free end of the blade. The third blade was damaged with an extra hole of 8 mm diameter between the two cuts, while the fourth blade was damaged with two extra holes of the same diameter on either side of the middle cut. The choice of the type and number of damages was made in such a way so as to have a gradual increase of damages to the rotating blades. Figure 10b presents the fourth rotating blade along with the introduced damages. As can be seen, the damage is restricted to one half side of the blade, while the Metglas ribbons to the other undamaged half. This was carried out on all rotating blades.

**Figure 11:**
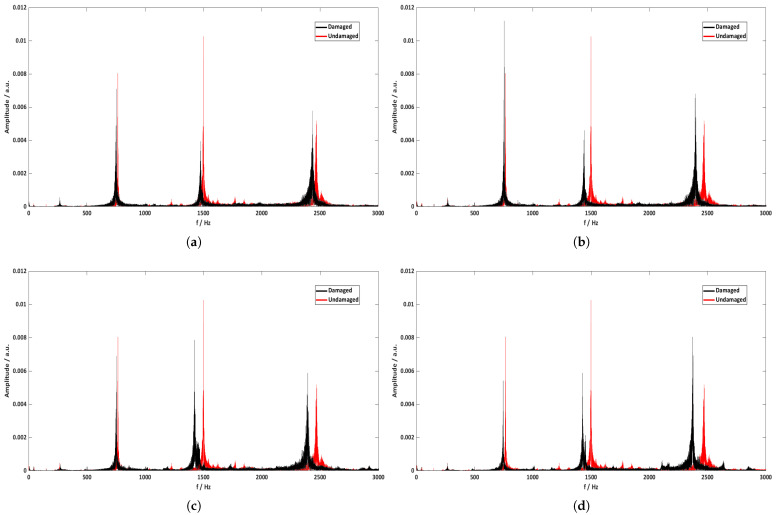
FFT spectrum results for each blade between the undamaged and the damaged state: (**a**) 1st, (**b**) 2nd, (**c**) 3rd, and (**d**) 4th.

Figure 11 shows the FFT graphs between the undamaged and damaged state of each rotating blade. These data were recorded in situ by the experimental method described in Section 3, and were collected and separated per blade through an algorithm in the Matlab software program. At first glance, it seems that the peaks of the bending modes are shifting to lower frequencies, and this can be confirmed in Table 6, where the frequency percentage differences between damaged and undamaged blades are presented. It is clear that as the magnitude of the damage is scaling from the first to the fourth blade, the percentage difference on each bending mode is increasing. This result is in line with the theory that the appearance of damages on beam-like structures strongly affects the stiffness of the beam, which is inversely proportional to the frequency. In addition, according to the results of Table 6, the bending mode that was changed the most is the fourth one for the fourth blade, with the percentage difference value being 7.2%. In general, the magnitude of change on each bending mode is directly related to the location where the damage occurs. Damages close to the nodes of the beam do not alter the frequency values of the corresponding mode as these are stationary points. On the other hand, damages located symmetrically between two nodes most alter the frequency values of the corresponding mode due to the highest amplitude of vibration at these points. Thus, it is very important to record data from different bending modes of each rotating blade in order to avoid false signals where the size of the damage can be large and the corresponding change in frequency very small, and vice versa.

## 5. Conclusions

In the current study, magnetoelastic ribbons are investigated as vibration sensors for real-time health monitoring of rotating aluminum blades. The ribbons are made by the Metglas alloy 2826 MB, and double-sided tape was used to attach them on one side of the beam blades. Coils were used in the detecting method to record in a contactless fashion, by means of Faraday’s law of induction, the vibration signal from the blades, as they were mechanically excited during rotation. The spectrum of the recorded signal was then retrieved using the fast Fourier transform (FFT) method. The FFT spectrum revealed five dominant peaks in the frequency range of 1–3 kHz that correspond to the first five bending modes of the rotating blades. The FFT peaks were identified with the use of the finite element commercial software ANSYS, in which the blades were simulated and the harmonics were determined numerically. The results showed a difference of less than 1% between the ANSYS and experimental bending modes, thus demonstrating the sensor’s sensitivity and accuracy in detecting and transmitting the vibrational state of the revolving blades.

Fixed damages were introduced to the rotating blades in order to test the sensor’s ability to detect changes in the blades’ vibration behavior. Side cuts and holes were the two types of damages introduced in a scaling manner, with the first blade having only one side cut and the last blade having two side cuts and two holes. When compared to the original state, which was the undamaged state, the results revealed a shift in the frequency values of the bending modes of each rotating blade, with the magnitude of the shift increasing from the first to the last blade. Based on these findings, it is concluded that these vibration sensors are excellent candidates for contactless and nondestructible evaluation of the mechanical health of rotating mechanical structures.

## Figures and Tables

**Table 1:** Physical and geometrical properties of the Metglas 2826 MB ribbons.

Sr. No.	Property	Value
1	Width	6 mm
2	Thickness	29 μm
3	Material Density	7.90 gr/cm^3^
4	Modulus of Elasticity	100–110 GPa
5	Average Stoichiometry	Fe_40_Ni_38_B_18_Mo_4_
6	Saturation Magnetostriction $\lambda_{s}$	12 ppm
7	Annealed Saturation Induction	0.88 T
8	Curie Temperature	353 °C
9	Crystallization Temperature	410 °C

**Table 2:** Physical properties of 3M^TM^VHB^TM^ 4930 double-sided tape.

Sr. No.	Property	Value
1	Adhesive Type	Modified Acrylic Adhesive
2	Tape Thickness	0.64 mm
3	Peel Adhesion	350 N/mm
4	Normal Tensile	1.1 MPa
5	Dynamic Shear	620 kPa
6	Static Shear	1.5 kg (22 °C)

**Table 3:** Physical and geometrical properties of the 6063 aluminum alloy rotating blades.

Sr. No.	Property	Value
1	Length	300 mm
2	Width	30 mm
3	Thickness	4 mm
4	Material Density	2.69 gr/cm^3^
5	Modulus of Elasticity	68.3 GPa

**Table 4:** Physical and geometrical properties of the detection coil.

Sr. No.	Property	Value
1	Coil Diameter	29.74 mm
2	Wire Diameter	0.16 mm
3	Tread Number	625
4	Wire Material	Cu
5	Electrical Resistance	34.9 Ω
6	Coefficient of Inductance	3.2 mH

**Table 5:** Comparison results between the ANSYS and the experimental frequency values.

Bending Modes	1st	2nd	3rd	4th	5th
ANSYS (Hz)	43.7	273.5	765.3	1498.2	2473.5
EXP (Hz)	43.3	267.3	765.4	1498.1	2468.7
Error (%)	0.92	2.32	0.01	0.01	0.19

**Table 6:** Frequency comparison results between the undamaged and the damaged state of each rotating blade.

Bending Modes		1st	2nd	3rd	4th	5th
Undamaged (Hz)		43.3	267.3	765.4	1498.1	2468.7
1st Blade	Damaged (Hz)	43.2	265.4	754.7	1467.2	2429.3
	Diff (%)	0.2	0.7	1.4	2.1	1.6
2nd Blade	Damaged (Hz)	42.6	261.4	751.6	1437.5	2395.1
	Diff (%)	1.6	2.2	1.8	4.0	3.0
3rd Blade	Damaged (Hz)	41.7	260.5	749.5	1421.8	2392.3
	Diff (%)	3.7	2.5	2.1	5.1	3.1
4th Blade	Damaged (Hz)	41.5	259.2	742.8	1390.1	2369.6
	Diff (%)	4.2	3.0	3.0	7.2	4.0

## Data Availability

Data available on request from the authors.
